# The diagnosis and subclassification of heart failure with preserved ejection fraction: a review

**DOI:** 10.1007/s10741-026-10618-2

**Published:** 2026-03-27

**Authors:** D. Creegan, R. C. Starling, A. Hameed, M. J. Daly, James O’Neill

**Affiliations:** 1 School of Medicine, RCSI University of Medicine and Health Sciences, Dublin, Ireland; 2 Department of Cardiovascular Medicine, Heart, Vascular and Thoracic Institute, Kaufman Centre for Heart Failure Treatment and Recovery, Cleveland Clinic, Cleveland, OH USA; 3 Tissue Engineering Research Group (TERG), Department of Anatomy and Regenerative Medicine, RCSI University of Medicine and Health Sciences, Dublin, Ireland; 4 Connolly Hospital, Blanchardstown, Dublin, Ireland

**Keywords:** HFpEF, Phenotypes, Diastolic dysfunction, Subclassification, Diastolic heart failure, Pathophysiology

## Abstract

The incidence and prevalence of Heart Failure with Preserved Ejection Fraction (HFpEF) continues to rise and has a mortality rate comparable to that of Heart Failure with Reduced Ejection Fraction (HFrEF). Whilst there is general agreement on the diagnostic criteria for HFrEF, the diagnostic criteria for HFpEF vary significantly which has resulted in inconsistent and varying inclusion criteria in clinical trials for this challenging syndrome. The HFpEF population is diverse and heterogeneous and various schemes of subcategorisation have been proposed. Failure of treatment trials to date can be explained, in part, by failure to consider the underlying sub-phenotypes within the HFpEF population. This review discusses the diagnostic uncertainty surrounding HFpEF and details the historical progression in the understanding of diastolic dysfunction and its role in the heart failure syndrome. An analysis of diagnostic criteria and suggested phenotypic subcategorisation is accompanied by a discussion of echocardiographic and invasive haemodynamic criteria. A detailed summary of the varied inclusion criteria and definitions employed in recent clinical trials is provided.

## Introduction

Heart Failure (HF) is defined as “a clinical syndrome with symptoms and/or signs caused by a structural and/or functional cardiac abnormality and corroborated by elevated natriuretic peptide levels and/or objective evidence of pulmonary or systemic congestion.” HF, in turn, is classified based on Ejection Fraction (EF). Heart Failure with Preserved Ejection Fraction (HFpEF) refers to the clinical syndrome of HF with an EF of ≥ 50% (1). The incidence and prevalence of HFpEF continue to rise, explainable in part by rising age, and increasing burdens of obesity with associated co-morbidities (2). HFpEF is associated with a significant reduction in quality of life, with the cardinal symptoms of dyspnoea and exercise intolerance largely attributable to systemic congestion. It now accounts for over half of all heart failure hospitalisations (3). Across four community-based cohorts in the US, the incidence rate was estimated to be 27 cases per 100 person years (4). Notably the all-cause mortality rates are comparable between HFrEF (Heart Failure with Reduced Ejection Fraction) and HFpEF, with studies suggesting a 5-year mortality rate as high as 75.7%. Non-cardiovascular causes of death remain higher in the HFpEF population (5). Hospitalisation rates for all categories of heart failure appear to be increasing over time. Based on the Nationwide Inpatient Sample (NIS) data, HFpEF accounted for 30.8% of all heart failure admissions with 23.3% having an unspecified EF (5).

Despite its rising prevalence and incidence, clinical trials have differed in their definitions of HFpEF and associated inclusion criteria. This review will begin by examining the evolving definitions of HFpEF, followed by a detailed analysis of the diagnostic challenges and their implications, particularly concerning clinical trials in this domain. It will then provide an overview of current approaches to subclassifying HFpEF.

## Methodology

This review was conducted following a detailed literature search using PubMed to identify key sources. The search was limited to articles published in English. No date restrictions were imposed to ensure inclusion of both foundational and recent studies. The selection of literature prioritised landmark clinical trials and high-quality consensus documents from recognised professional societies. Review articles, meta-analyses, randomised controlled trials, observational studies and position statements were all considered. Given the narrative nature of this review, findings from individual studies were qualitatively synthesised rather than subjected to formal meta-analytic techniques. While the review aimed to be comprehensive, it is not a systematic review and thus may be subject to selection bias inherent in narrative reviews.

### Defining HFpEF

The concept of a heart failure syndrome in the setting of normal left ventricular ejection fraction was first described in 1982. Luchi et al. measured left ventricular ejection fraction in 62 elderly patients using gated wall motion (6). They identified a subset of patients with clinical evidence of heart failure with a normal ejection fraction. They hypothesised that diastolic dysfunction must explain the clinical syndrome. This condition was initially referred to as “Diastolic Heart Failure”.

A subset of patients with diastolic dysfunction was recognised in an elderly hypertensive cohort in the mid-1980s. The predominately female population had evidence of prolonged early diastolic filling and reduced peak diastolic dimension increase. This was associated with the clinical presentation of heart failure and represented, at the time, a unique subset of heart failure patients (7). Cohn et al. described a cohort of patients with heart failure despite a normal Ejection Fraction in 1990, describing a multifactorial pathophysiology. The condition was described as “a more benign condition” than HFrEF (8). The concept of diastolic heart failure was further described by Brutsaert et al. in 1997, defined as an increase in left ventricular filling pressure, leading to symptoms and signs of congestion and an upward shift of the left ventricular pressure-volume relationship in diastole (9).

Three criteria for diagnosis were proposed by a working group of the European Society of Cardiology in 1998. These included the presence of signs or symptoms of congestive heart failure, the presence of normal left ventricular systolic function and the presence of abnormal left ventricular relaxation (10). As noted by Zile et al., this approach has been criticised for a number of reasons (11). Firstly, it was argued that measurements of ventricular relaxation are load dependent. This leads to poor sensitivity and specificity of this measurement, limiting its usefulness in the clinical setting. Secondly, the core symptoms of congestion lack specificity for the heart failure phenotype. Thirdly, normal left ventricular ejection fraction was defined as greater than or equal to 45%. This arbitrary cutoff value lacked correlation with underlying pathophysiological features and disease trajectory. It did not consider LV volumes, concentric remodelling and the presence or absence of increased filling pressures or abnormalities in relaxation and distensibility. Indices such as LV long axis functional reserve and peripheral blood flow have stronger correlations with exercise capacity than an arbitrary left ventricular ejection fraction value (12). With these criticisms in mind, these criteria were refined by Vasan and Levy (13). They recommended a diagnostic algorithm that included definite, probable and possible “diastolic heart failure”. Definite “diastolic heart failure” required evidence of systemic congestion, a left ventricular ejection fraction of greater than 50% within 72 h of the purported heart failure event, and evidence of diastolic dysfunction on cardiac catheterisation. In later studies the requirement for diastolic dysfunction on invasive assessment was removed. Zile et al. described diastolic heart failure simply as a clinical syndrome with symptoms and signs of congestion and a normal ejection fraction (14).

The term “Diastolic Heart Failure” was subsequently replaced with HFpEF, as seen in the ACC/AHA 2005 guideline update on the diagnosis and management of chronic heart failure in the adult (15). Importantly, the condition can occur in the absence of diastolic dysfunction and the presence of diastolic dysfunction can occur in the absence of HFpEF. Recent efforts have resulted in a consensus definition comprising of: a left ventricular ejection fraction of ≥ 50% with elevated BNP (B-Type Natriuretic Peptide) or other evidence of congestion (2). An over-reliance on BNP for diagnosis of HFpEF has the potential to underestimate prevalence and contributes to under-diagnosis in certain patient populations, particularly those with obesity, who tend to have lower BNP levels (2). It has been recommended that a correction factor be applied to BNP levels in patients with obesity when using BNP cut-off values as inclusion criteria for clinical trials (16). In contrast, patients with atrial fibrillation tend to have higher baseline BNP levels compared to their counterparts without. Given the high prevalence of obesity and atrial fibrillation in the HFpEF population, interpretation of the significance of a single BNP level can be challenging (17).

Several clinical scores have been developed that can be used to diagnose HFpEF. The most cited score is the H_2_FPEF score. The score is composed of several components, as illustrated in Table 1. The final score indicates the likelihood of HFpEF. For those patients who score in the indeterminate range, further haemodynamic testing is recommended (18). Studies have demonstrated the potential usefulness of the H_2_FPEF score as a risk prediction tool. A retrospective cohort study of a HFpEF population found a sensitivity and specificity of 68.3% and 55.4% respectively for all-cause mortality using a cut-off H_2_FPEF score value of 5.5 (19). The absence of BNP in the clinical variables is notable, potentiating the usefulness of the score in patients presenting with signs and symptoms of congestion alone. However, given the low specificity of key signs and symptoms of congestion for the heart failure syndrome, this use may be overstated. For example, a 65 year old obese male with atrial fibrillation on two antihypertensives presenting with symptoms or signs of congestion has a > 90% probability of having HFpEF, regardless of BNP level. Clearly, potential mimics, both cardiac and non-cardiac, that may account for the symptom presentation must also be considered - one can easily see how, in an overweight person with an elevated BNP and breathlessness suffering from a pulmonary embolus, using the simple diagnostic criteria as above, they could be mis-labelled as having HFpEF.

**Table 1:** Components of the H_2_FPEF score

Clinical Variable	Points	Score
BMI > 30 kg/m2	2	Score of 3 > 50% probabilityScore of 4 > 70% probabilityScore of 5 > 80% probabilityScore of 6 > 90% probability
Atrial Fibrillation	3	
Age > 60	1	
Treatment with 2 or more antihypertensive agents	1	
E/e’ ratio of > 9	1	
Pulmonary Artery Systolic Pressure of > 35mmHg	1	

The HFpEF-ABA score is the most recent tool that has been developed to facilitate diagnosis. Reddy et al. designed this score to simplify the detection of patients with a high likelihood of developing HFpEF (20). Their score employs three components: Age, BMI and presence of atrial fibrillation. Patient specific data is entered into a complex mathematical formula to derive likelihood ratios for the presence of HFpEF. Further studies examining the validity of this score are needed (18).

Both the H_2_FPEF and HFpEF-ABA scores have demonstrated independent association with peak Pulmonary Capillary Wedge Pressure (PCWP) and Pulmonary Artery Pressure (21). In the same study, higher scores in both models were associated with greater impairment in key measures of disease severity (21).

In 2019, a consensus recommendation from the Heart Failure Association of the European Society of Cardiology was published. The revised recommendations have incorporated more robust clinical, imaging and lab-based criteria into a stepwise diagnostic approach (12). The new recommendations also attempt to avoid including or excluding a diagnosis of HFpEF based on arbitrary cut off values associated with diagnostic tests. The guideline advises a stepwise approach to the diagnosis of HFpEF, acknowledging the complexity of patient presentations. Step 1 involves an initial assessment of the pre-test probability. A detailed assessment of patient demographics, comorbidities and symptoms and signs should ensue. This can be supplemented by basic ECG and basic initial echocardiographic criteria. Step 2 then involves a detailed diagnostic workup, consisting of extensive echocardiography and laboratory analysis, focusing mainly on BNP or NT-pro-BNP. The role of natriuretic peptides here is challenging. Patients with HFpEF often have lower levels than patients with reduced ejection fraction, despite comparable congestion (2). Step 3 focuses on advanced diagnostic investigations, particularly relevant where diagnosis remains uncertain. Diastolic stress testing and stress echocardiography can be considered at this stage in the process. However, it must be noted that these are rarely performed in clinical practice. The final step considers an aetiological work-up. This is the first occasion in which a society guideline has identified the importance of sub-classifying HFpEF into different categories based on aetiological and pathophysiological mechanisms.

**Fig. 1:**
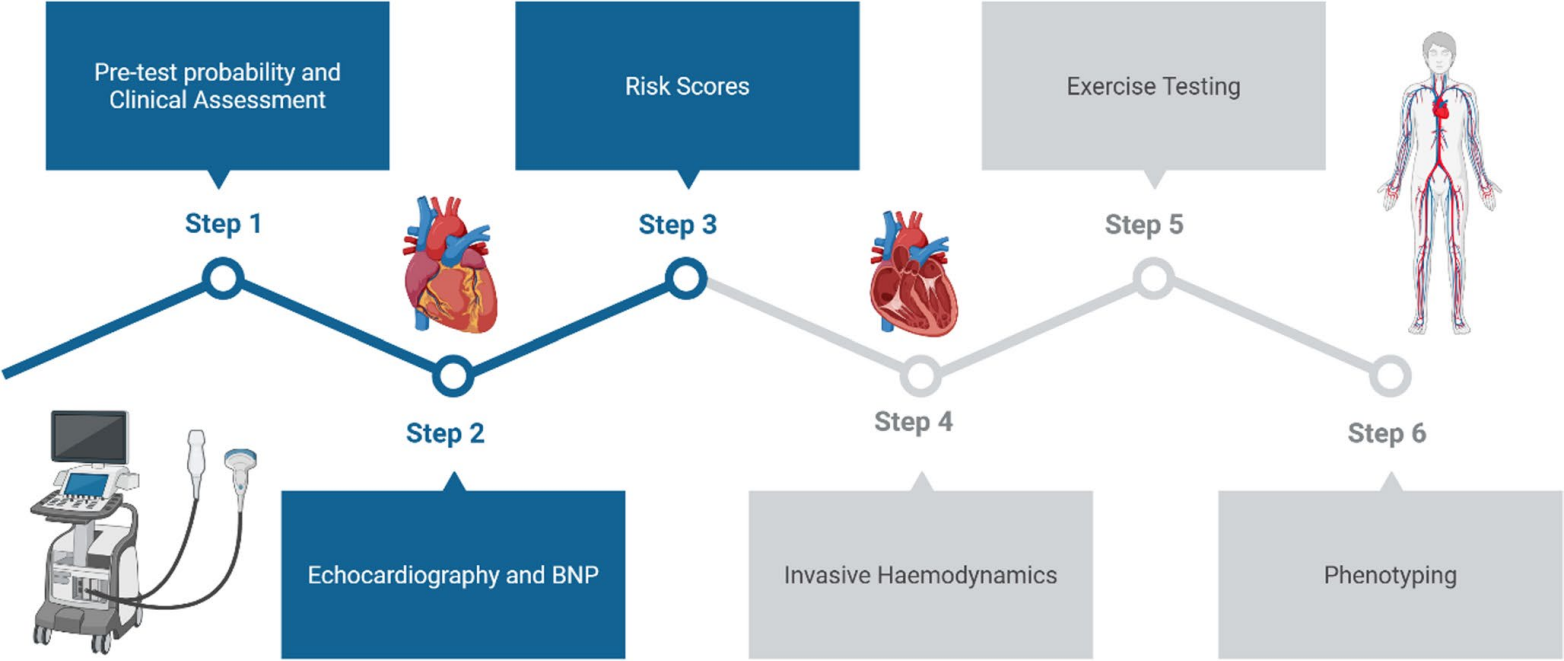
Proposed diagnostic approach for HFpEF

More recently, the American College of Cardiology published a consensus recommendation in 2023. Accepting that the diagnosis of HFpEF remains difficult due to the absence of characteristic structural and functional abnormalities, the H_2_FPEF and the HFA-PEFF diagnostic scoring systems are endorsed. Given the difficulties with implementing the HFA-PEFF algorithm in clinical practice, the H_2_FPEF score is put forward as the more useful clinical approach in everyday practice (22). Taking all this into account, Fig. 1 outlines a proposed diagnostic approach.

### HFpEF mimickers

Exclusion criteria are also of significant importance when considering a diagnosis of HFpEF. It is not uncommon for individuals to be falsely labelled as having HFpEF when an alternative condition such as coronary artery disease, amyloidosis, hypertrophic cardiomyopathy or pulmonary hypertension is the cause of the patient’s presentation. Heart failure can, of course, co-exist with these diagnoses, contributing further to the complexity. Importantly, inclusion of such patients in HFpEF trials dilutes any potential treatment effects and, in clinical practice, misdiagnosis results in denial of alternative disease-oriented therapies that may improve prognosis, of particular relevance for amyloidosis. The potential for cardiac amyloidosis (both AL and ATTR) to masquerade as a HFpEF phenotype can be seen in a screening study from 2015 which identified increased cardiac uptake in 99Mtc-DPD scanning in 13% of HFpEF patients (23). A similar study in 2016, using multi-modality imaging identified a prevalence of 18% for ATTR-amyloid and 10% for AL amyloid in an undifferentiated HFpEF population (24).

### Attempts to subclassify HFpEF

The heterogeneity of the HFpEF population represents a significant challenge when it comes to research and clinical practice. The development of a clear understanding of this heterogeneity is essential for advancing clinical trial design. The diversity of the population is seen in its comorbidity profile. Patients with HFpEF frequently have multiple comorbidities with the most common being hypertension, coronary artery disease, diabetes mellitus, chronic kidney disease, atrial fibrillation and obesity (25). Newer diagnostic criteria are incorporating the significance of underlying phenotypes of HFpEF when considering a diagnosis. The underlying phenotype affects the underlying pathophysiology and the progression of the disease. Numerous subclassification paradigms have been suggested. There is, however, significant methodological variation across studies. One such approach proposes classifying HFpEF based on the presence of absence of hypertension. Management should be tailored to the underlying pathophysiological mechanisms. Aggressive treatment of hypertension may prevent the progression of HFpEF in the hypertension subtype while the non-hypertension subtype is often secondary to other treatable conditions such as valvular disease (26).

Given the significant variability within the HFpEF population, the task of identifying criteria for sub-categorisation via the traditional human-led hypothesis method has proved challenging. In addition, significant overlap exists between different phenotypes, making clean and neat sub-categorisation cognitively difficult. It is within this environment that machine learning has arisen as a viable mechanism for identifying trends amongst HFpEF patients that may suggest an underlying phenotypic sub-classification (27). Table 2 highlights the phenotypes that have been elucidated in key trials to date.

**Table 2:** Characteristics of each HFpEF phenogroup in key studies

	Shah et al. (28)	Sotomi et al. (30)	Makino et al. (32)
Phenogroup 1	Younger patients, moderate diastolic dysfunction	Cardiac rhythm disorders with high burdens of A. Fib	Younger patients with metabolic disorders
Phenogroup 2	Obesity, diabetes, OSA and significantly impaired relaxation	High prevalence of HTN and ventricular-arterial coupling failure	Systemic congestion and raised BNP levels
Phenogroup 3	Older patients with CKD and electrical and myocardial remodelling	Low output and significant congestion	Multiple comorbidities, particularly vascular
Phenogroup 4		Systemic Failure	Older patients with arrhythmias

Shah et al. attempted to sub-classify HFpEF with the use of machine learning and cluster analysis (28). Four hundred and twenty ambulatory patients were recruited from outpatient clinics and followed prospectively. Eligibility was restricted to patients with symptomatic heart failure with an EF of greater than 50%. Additional markers of HFpEF such as grade 2 or 3 diastolic dysfunction on echocardiography, elevated LV filling pressures on invasive haemodynamic monitoring or elevated BNP were identified as inclusion criteria. The modern diagnostic algorithms discussed above were not used. Using unsupervised deep learning algorithms, the authors succeeded in categorising their dataset into 3 distinct phenotypes. Each phenotype shared common characteristics and followed a separate, independent outcome trajectory. The three phenotypes were as follows: (1) patients of younger age with normal BNP but evidence of moderate diastolic dysfunction on echocardiography. (2) Patients with obesity and diabetes with a high prevalence of obstructive sleep apnoea (OSA) and significantly impaired relaxation. (3) Patients of older age with chronic kidney disease (CKD) and electrical and myocardial remodelling, pulmonary hypertension and right ventricular dysfunction.

Early approaches focused on the use of continuous variables in discrete categories, potentially excluding important discriminating features. Segar et al. addressed this problem with the use of an updated unsupervised cluster analysis of data from the TOPCAT trial (29). This analysis included both continuous and categorical data. The authors chose to restrict their analysis to participants from the Americas. This was due to reported variation in co-morbidities, presentation and even response to therapy across the geographical spectrum. The key discriminatory features across subgroups in the TOPCAT analysis included serum glucose, bilirubin, diabetes status, Body Mass Index (BMI), interventricular conduction delay and left ventricular function.

Latent-class analysis was used by Sotomi et al. to subdivide HFpEF patients in the acute setting. The patient cohort was derived from the PURSUIT HFpEF study (30). Based on the concept of cluster analysis used in a variety of disparate fields, latent class analysis allows direct modelling of variables to identify trends within the dataset (31). These trends ultimately form subtypes or subgroupings within the dataset. Based on their analysis, the authors were able to sub-classify acute HFpEF into four distinct phenotypes. These phenotypes differ from those identified by Shah et al. It is possible that the differences in baseline population characteristics (i.e., acute heart failure in Sotomi’s study and chronic heart failure in Shah’s seminal machine learning study) can account for this divergence. The first subgroup had a high burden of “rhythm trouble”, most notably atrial fibrillation. Comorbidities were less frequent in this group and plasma volume expansion less of a prominent issue. The second group had a high prevalence of hypertension with classic diastolic dysfunction present on echocardiography. The authors describe this group as consisting of those with “ventricular-arterial coupling failure.” The third group had evidence of low output and significant congestion. This group was associated with the poorest prognosis overall. The final group was characterised as the “systemic failure” cohort, comprising patients with elevated CRP levels and evidence of infectious triggers contributing to their decompensation.

Latent class analysis has also been used to evaluate associations between phenotypes and clinical outcome. A study published in 2024 applied the statistical method to 1,281 patients with HFpEF who were admitted with decompensation (32). Four phenotypes were identified which were largely similar to the phenotypes identified in the earlier studies by Shah et al. The first phenotype included younger patients with metabolic disorders. The second phenotype included patients with systemic congestion and raised BNP levels. The third phenotype had multiple comorbidities, most notably vascular disease. The fourth phenotype included older patients with bradyarrhythmia or atrial fibrillation. After adjusting for age and sex, the second phenogroup had the highest risk of all cause death and cardiac death. Phenotype specific treatment strategies were suggested. For phenotype 1, which consisted of younger patients with obesity and metabolic disorders, weight loss was considered the mainstay of treatment. In addition, SGLT2 inhibitors may be a useful adjunct in this population. For phenotype 2, diuretics were considered the most appropriate intervention. Phenotype 3 was associated with a high burden of vascular comorbidities, indicating that treatment should prioritise the management of these underlying conditions. For phenotype 4, a rhythm control strategy targeting atrial fibrillation should be considered (32).

Although these studies suggest potential utility of employing a machine-learning based subclassification system for HFpEF, further work is required in this area. There remains significant heterogeneity between clusters depending on individual study. In addition, these clusters have not been externally validated and the way these subtypes can translate into routine clinical decision making remains to be seen.

### Echocardiography

Key echocardiographic criteria used in the diagnosis of HFpEF are outlined in Table 3. Septal and lateral mitral annular peak early diastolic velocity or e’ is considered first. The main determinant of e’ is left ventricular relaxation. Cut-off values for e’ are adjusted for age in the ESC guideline with values tending to decline with age (33). Mean Pulmonary Capillary Wedge Pressure (PCWP) is represented by average septal-lateral E/e’ ratio. It correlates well with degrees of fibrosis and stiffness of the left ventricle (34). Tricuspid regurgitation peak velocity or Pulmonary Arterial Systolic Pressure (PASP) is the next criterion considered. Rising tricuspid regurgitation peak velocities are indicative of increasing PASP and are an indirect marker of diastolic dysfunction (35). The role of left ventricular global longitudinal systolic strain analysis is being increasingly recognised. Strain values have been shown to correlate with NT-Pro BNP levels (36) and impairments in strain are also predictive of heart failure hospitalisation events (37). The final parameter is Left Atrial Volume Index (LAVI). Co-morbidities must be taken into consideration when analysing LAVI as those with atrial fibrillation have larger LAVI measurements, regardless of the presence of HFpEF (38).

**Table 3:** Key echocardiographic parameters in the diagnosis of HFpEF

Index	Description	Reflects	Pitfalls
e’	Septal and lateral mitral annular peak early velocity	LV relaxation	Values tend to decline with age
E/e’	Ratio between early mitral inflow velocity and mitral annular early diastolic velocity	Mean Pulmonary Capillary Wedge Pressure; Correlates with stiffness of left ventricle	E’ values decrease with age
Tricuspid Regurgitation Peak Velocity	Doppler assessment of the peak Tricuspid Regurgitation velocity	Pulmonary Artery Systolic Pressure	Dependent upon acquisition of a clear Tricuspid Regurgitation envelope
Left ventricular strain	Measure of left ventricular deformation	Left ventricular stiffness; Correlates with NT-pro-BNP levels	
E/A ratio	Ratio of early diastolic flow velocity divided by late diastolic transmitral flow velocity	Diastolic dysfunction; LV remodelling	Not useable in atrial fibrillation
LAVI	Left Atrial Volume Index	Diastolic dysfunction	Those with atrial fibrillation have larger LAVI measurements

LAVI; left atrial volume index, LV; left ventricular, BNP; B-type natriuretic peptide

Although rarely performed, stress echocardiography is useful in the diagnosis of HFpEF, particularly in patients in whom uncertainty regarding the diagnosis persists. As the heart rate increases with exercise, filling times are reduced. The normal heart will compensate by an increased reliance on early diastolic suction for filling. In patients with diastolic dysfunction, this early diastolic suction is impaired. Increased left ventricular filling pressures are then needed to maintain adequate cardiac output to meet the increased metabolic demand. Hence, filling pressures may not be evident at rest but become apparent with exercise (18). The key index measured during exercise echocardiography is the E/e’ ratio. An E/e’ ratio of ≥ 15 is abnormal. Peak Tricuspid Regurgitation (TR) velocity can also be used, with a value greater than 3.4 m/s considered abnormal (18). The ESC HFA-PEFF score considers both indices in its algorithm. Other useful indices include E/A ratio, isovolumetric relaxation time, left atrial volume and pulmonary vein flow velocities (18).

Tricuspid Regurgitation (TR) warrants special mention. TR and HFpEF commonly co-exist. One study showed that up to 90% of patients with Tricuspid Regurgitation (TR) had evidence of HFpEF, as diagnosed using the H_2_FPEF score (40). There are several pathophysiological interactions between HFpEF and TR. Severe TR may result in right ventricular dilatation with resulting ventricular interdependence as the ventricular septum bulges into the left ventricle. This then leads to impaired left ventricular filling and diastolic dysfunction. Alternatively, severe TR may be the result of elevated left sided pressures and Group 2 pulmonary hypertension from underlying HFpEF (40). Atrial fibrillation, which is prevalent in the HFpEF population (39) and associated with atrial remodelling and annular dilatation may also account for the high prevalence of TR (41). It is possible therefore that a subset of severe TR patients may meet the diagnostic criteria for HFpEF. Notably, patients with severe TR are often excluded from HFpEF trials.

### Invasive Haemodynamics

Although performed less frequently in practice, HFpEF is associated with characteristic abnormalities on invasive haemodynamic assessments. Invasive haemodynamics are particularly useful when the patient has an intermediate probability of HFpEF based on validated scoring tools such as the HFA-PEFF score. A study of 2550 patients confirmed the additional prognostic utility of invasive testing of left ventricular end-diastolic pressure in patients with an intermediate HFA-PEFF score (42). In individuals with obesity, invasive testing has also been shown to be of significant benefit. It is more difficult to discriminate between the presence or absence of HFpEF in the obese population due to lower baseline BNP levels. Characteristic abnormalities on echocardiography are also less common in this population (43).

A recent Scientific Statement from the American Heart Association provides an overview of the invasive haemodynamic criteria that support a diagnosis of HFpEF (44). These criteria are outlined in Table 4. Similar to findings in echocardiography, measurements can be normal at rest, manifesting abnormality with exercise. Studies have shown prominent increases in Pulmonary Capillary Wedge Pressure (PCWP) and higher left sided filling pressures during exercise in patients with HFpEF (45). HFpEF is also associated with the development of exercise-induced pulmonary hypertension with significant increases in Pulmonary Artery Pressure (PAP). A study by Borlaug et al. demonstrated that 88% of HFpEF patients who underwent invasive haemodynamic monitoring during exercise met the criteria for exercise induced pulmonary hypertension (45).

**Table 4:** Invasive haemodynamic criteria in the diagnosis of HFpEF

Measure	Criteria
Resting PAWP	≥ 15mmHg
PAWP with fluid bolus or passive leg raise	≥ 20mmHg
PAWP during supine exercise	≥ 25mmHg
PAWP during upright exercise	≥ 20mmHg
PAWP/CO slope	> 2mmHg/L/min

PAWP: pulmonary arterial wedge pressure, CO: cardiac output

An understanding of exercise haemodynamics in HFpEF has led to novel approaches to therapy, most notably the interatrial shunt to unload the left heart during exercise. Studies have shown a 3-5mmHg decrease in PCWP during exercise. Demonstrating the importance of haemodynamic assessments in the classification of HFpEF, those with a lower Pulmonary Vascular Resistance (PVR) responded more favourably (46).

### Clinical Trials in HFpEF

Despite attempts to standardise the diagnostic parameters for HFpEF, trials in HFpEF have been inconsistent in applying diagnostic criteria. There is significant variability in the inclusion criteria used in key HFpEF trials. This limits the ability to compare results and reduces the overall clinical applicability. Table 5 illustrates the varying definitions employed in key recent HFpEF trials.

**Table 5:** Variations in definitions of HFpEF in clinical trials

Trial	LV EF	NT-Pro BNP (pg/ml)	Structural Abnormality	Signs/Symptoms
CHARM – Preserved (2003) (47)	> 40%	N/A	N/A	NYHA II-IV
I-PRESERVE (2008) (48)	≥45%	N/A	Left atrial enlargement LVH	NYHA II-IV
PARAGON-HF (2019) (49)	≥45%	> 300 or > 900 if A Fib	Left atrial enlargement LVH	NYHA II-IV
TOPCAT (2014) (50)	≥45%	> 360	N/A	One or more prespecified symptoms
EDIFY (2017) (51)	≥ 50% (changed to 45% after 9 months)	> 300 (changed to 220 after 9 months)	E/e’ ratio > 13 e’ lateral < 10 cm/sec e’ septal <8 cm/sec LAVI > 34 ml/m2	NYHA II-III
EMPEROR PRESERVED (2021) (52)	> 40%	> 300 or > 900 if A Fib	Left atrial enlargement LVH	NYHA II-IV
PURSUIT HFpEF (2020) (53)	≥ 50%	> 400	N/A	N/A
DELIVER (2022) (54)	> 40%	> 300 or > 600 if A Fib	Left atrial enlargement LVH	NYHA II-IV
INDIE HFpEF (2018) (55)	≥ 50%	> 400	E/e’ >/=15 Left atrial enlargement	NYHA II-IV
PARABLE (2023) (56)	> 50%	> 100	LAVI > 28 ml/m2	N/A
MyPACE (2023) (57)	> 50%	> 400	Diastolic dysfunction or LVH or left atrial dilatation	NYA > II
STEP HFpEF (2023) (58)	≥ 45%	Stratified based on BMI	LVH, left atrial enlargement, septal e’ <7 cm/sec or lateral e’ <10 cm/sec or Average E/e’ >/=15, PASP > 35mmHg	NYHA II-IV
STEP HFpEF DM (2025) (59)	≥ 45%	Stratified based on BMI	Septal e’ < 7 cm/sec or lateral e’ < 10 cm/sec or average E/e’ >/= 15 Left atrial enlargement, LVH, PASP > 35mmHg	NYHA II-IV
PIROUETTE (2021) (60)	> 45%	>/=300 or >/=900 if A. Fib	Myocardial fibrosis on cardiac MRI	At least one symptom and one sign of Heart Failure
SERENADE (2025) (61)	≥ 40%	>/=200 or >/=500 if A. Fib	LVH, left atrial enlargement, LAVI >/= 28 ml/m2, Peak TR velocity > 2.8 m/s, TAPSE < 17 mm	NYHA II-III
PANACHE (2019) (62)	≥ 45%	>/=300 or >/=900 if A. Fib	Left atrial enlargement LVH Elevated filling pressures	NYHA II-IV
SUMMIT (2024) (63)	≥ 50%	> 200 or > 600 if A Fib	Left atrial enlargement or elevated left ventricular filling pressures	NYHA II-IV
RELAX (2013) (64)	≥ 50%	>/=400 (or evidence of elevated PCWP)	Left atrial enlargement	NYHA II-IV
PARAMOUNT (2013) (65)	≥ 45%	> 500	N/A	NYHA II-IV
FAIR HFpEF (2024) (66)	≥ 45%	> 300 or > 600 if A. Fib	E/e’ >13 or left atrial enlargement	NYHA II-III and treatment with diuretic
REGRESS HFpEF (2024) (results not yet published) (inclusion criteria as per ClinicalTrials.gov)	≥ 50%	BNP > 125 or 150 if A. Fib	Left atrial enlargement	NYHA II-IV
EASi HF Preserved (underway) (inclusion criteria as per ClinicalTrials.gov)	≥ 40%	Stratified based on BMI	LVH or left atrial enlargement	NYHA II-IV
REDEFINE HF (underway) (inclusion criteria as per ClinicalTrials.gov)	≥ 40%	> 500 or > 1500 if A. Fib	N/A	Heart Failure signs and symptoms
SOGALDI PEF (2024) (67)	> 40%	> 220 or > 660 if A. Fib	Left atrial enlargement or LVH or evidence of diastolic dysfunction	NYHA II-IV
FINEARTS-HF (2024) (68)	≥ 40%	>/= 300 or >/= 900 if A. Fib	Left atrial enlargement or LVH	NYHA II-IV
SPIRRIT HFpEF (2024) (69)	≥ 40%	> 300 or > 750 if A. Fib	N/A	NYHA II-IV
ALDO-DHF (2013) (70)	≥ 50%	N/A	Diastolic Dysfunction of at least Grade 1	NYHA II-III
CAPACITY HFpEF (2020) (71)	≥ 40%	> 300	LVH, Left atrial enlargement, E/e’ >15	NYHA II-IV
PARAGLIDE-HF (2023) (72)	> 40%	> 500 or > 1000 if A Fib	N/A	N/A
REDUCE LAP HF2 (2024) (73)	≥ 40%	> 150 or > 450 if A Fib	Left atrial enlargement, lateral e’ <10 cm/sec or septal e’ <8 cm/sec or lateral E/e’ >10 or septal E/e’ >15	NYHA II-IV

LVEF: left ventricular ejection fraction, LVH: left ventricular hypertrophy, NYHA: New York Heart Association, LAVI: Left atrial volume index. PCWP: pulmonary capillary wedge pressure

The table provides a detailed comparison of inclusion criteria across the most significant HFpEF clinical trials. The most notable area of variation lies in the left ventricular ejection fraction (LVEF) thresholds used to define preserved systolic function. Most trials set the cut-off at or above 40% to 50%, with some, such as CHARM Preserved, employing a > 40% threshold, while others like EDIFY and PURSUIT HFpEF, use a higher bar of ≥ 50%.

Clinical trials also vary in their requirements for structural heart disease markers. While many specify left atrial enlargement or left ventricular hypertrophy, some incorporate additional echocardiographic parameters such as E/e’ ratio and left atrial volume index (LAVI). For instance, the EDIFY trial requires an E/e’ ratio > 13 and specific lateral and septal e’ velocity criteria to confirm diastolic dysfunction, while others such as TOPCAT focus more generally on left atrial enlargement and LVH. Symptomatology is a common inclusion feature, usually defined by New York Heart Association (NYHA) functional classes II to IV. However, not all trials require this explicitly; some focus more on objective criteria like biomarker levels or structural abnormalities. Overall, this table underscores significant heterogeneity in trial inclusion criteria, highlighting the challenges in standardising patient cohorts for HFpEF research.

## Summary

This review highlights the diagnostic challenges and the diagnostic overlaps that mimic “HFpEF”. The population is heterogeneous with multiple comorbidities (e.g., obstructive sleep apnoea, chronic kidney disease, obesity, diabetes mellitus, atrial fibrillation, coronary artery disease) that influence prognosis and require independent therapeutic measures. Recent trials have demonstrated robust data indicating benefit with SGLT2 Inhibitors and Finerenone in this population. In addition, evidence for the benefit of GLP-1 Receptor Agonists is also compelling. Semaglutide has demonstrated clinically relevant improvement in functional outcomes and weight loss in the STEP HFpEF trials (52, 53). The benefit is also seen in patients who do not have obesity (74). In tandem with an ageing population and an increasing prevalence of obesity it is anticipated that the incidence of HFpEF will continue to increase. Although the diversity within the HFpEF population suggests multiple underlying pathophysiological mechanisms, the hypothesis of adipokine, “angry” visceral fat has recently been proposed as the unifying aetiology in most HFpEF phenotypes (75).

## Conclusion

**Fig. 2:**
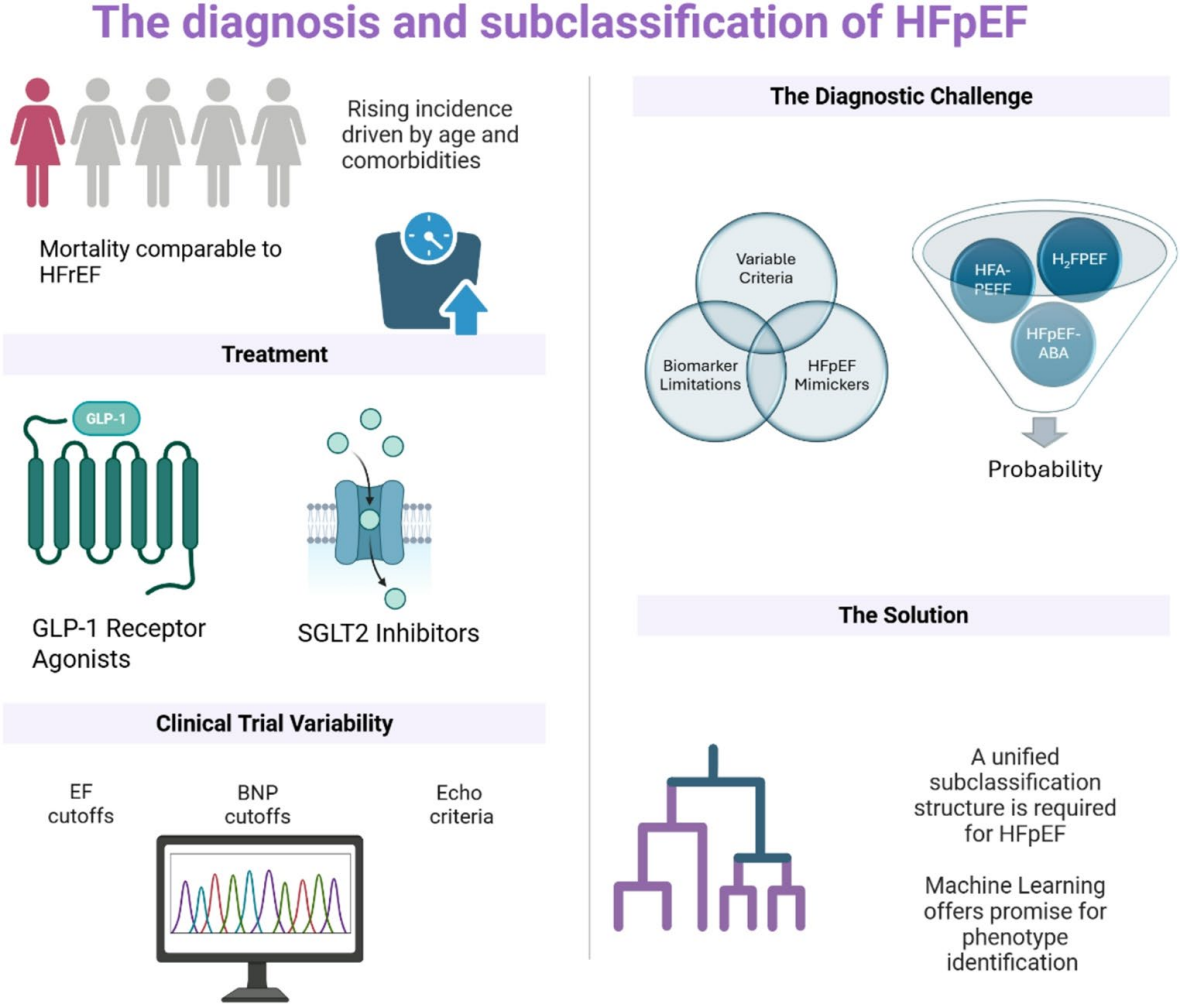
Central illustration

Despite the increasing prevalence, morbidity and mortality associated with HFpEF, diagnostic uncertainty remains. Does it really matter? Heart failure is a spectrum. Increasingly we are seeing pharmacologic therapies that span the ejection fraction spectrum (ARNi, SGLTi, beta blockers, MRA). Clinical trials have inconsistently defined “HFpEF” with differing inclusion criteria based upon LV EF. The HFpEF population is diverse and heterogeneous which complicates clinical trial design. Failure of treatment trials to date can be explained, in part, by failure to apply standardised definitions of HFpEF, and to consider the underlying sub-phenotypes within HFpEF. The success of future therapeutic trials rests on the identification of a clear diagnostic algorithm and a clear subclassification paradigm (Fig. 2).

## Data Availability

No datasets were generated or analysed during the current study.

## Abbreviations

ARNi: Angiotensin Receptor Neprilysin Inhibitor
BMI: Body Mass Index
BNP: B-Type Natriuretic Peptide
ECG: Electrocardiogram
EF: Ejection Fraction
HFpEF: Heart Failure with Preserved Ejection Fraction
HFrEF: Heart Failure with Reduced Ejection Fraction
LAVI: Left Atrial Volume Index
LV: Left Ventricle
LVH: Left Ventricular Hypertrophy
MRA: Mineralocorticoid Receptor Antagonist
NYHA: New York Heart Association Classification
PCWP: Pulmonary Capillary Wedge Pressure
PVR: Pulmonary Vascular Resistance
SGLT2i: Sodium Glucose Co-Transporter 2 Inhibitors
TR: Tricuspid Regurgitation
